# Comparative Analysis and Strength Estimation of Fresh Concrete Based on Ultrasonic Wave Propagation and Maturity Using Smart Temperature and PZT Sensors

**DOI:** 10.3390/mi10090559

**Published:** 2019-08-23

**Authors:** Najeebullah Tareen, Junkyeong Kim, Won-Kyu Kim, Seunghee Park

**Affiliations:** 1 Department of Civil, Architecture & Environmental System Engineering, Sungkyunkwan University, Gyonggi-do 16419, Korea; 2 Research Strategy Team, Advanced Institute of Convergence Technology, Gyonggi-do 16229, Korea; 3 Department of Convergence Engineering for Future City, Sungkyunkwan University, Gyonggi-do 16419, Korea; 4 School of Civil, Architectural Engineering & Landscape Architecture, Sungkyunkwan University, Gyonggi-do 16419, Korea

**Keywords:** nondestructive testing, maturity method, concrete early-age strength, SmartRock, ultrasonic waves, PZT (piezoelectric) sensors, structural health monitoring

## Abstract

Recently, the early-age strength prediction for RC (reinforced concrete) structures has been an important topic in the construction industry, relating to project-time reduction and structural safety. To address this, numerous destructive and NDTs (non-destructive tests) are applied to monitor the early-age strength development of concrete. This study elaborates on the NDT techniques of ultrasonic wave propagation and concrete maturity for the estimation of compressive strength development. The results of these comparative estimation approaches comprise the concrete maturity method, penetration resistance test, and an ultrasonic wave analysis. There is variation of the phase transition in the concrete paste with the changing of boundary limitations of the material in accordance with curing time, so with the formation of phase-transition changes, changes in the velocities of ultrasonic waves occur. As the process of hydration takes place, the maturity method produces a maturity index using the time-feature reflection on the strength-development process of the concrete. Embedded smart temperature sensors (SmartRock) and PZT (piezoelectric) sensors were used for the data acquisition of hydration temperature history and wave propagation. This study suggests a novel relationship between wave propagation, penetration tests, and hydration temperature, and creates a method that relies on the responses of resonant frequency changes with the change of boundary conditions caused by the strength-gain of the concrete specimen. Calculating the changes of these features provides a pattern for estimating concrete strength. The results for the specimens were validated by comparing the strength results with the penetration resistance test by a universal testing machine (UTM). An algorithm used to relate the concrete maturity and ultrasonic wave propagation to the concrete compressive strength. This study leads to a method of acquiring data for forecasting in-situ early-age strength of concrete, used for secure construction of concrete structures, that is fast, cost effective, and comprehensive for SHM (structural health monitoring).

## 1. Introduction

Concrete is an extensively used material in construction owing to its rich qualities, including being cost-effective, durable, and moldable into shapes, permitting the construction of safe and reliable structures. By contrast, the complex mixture (cement, sand, aggregates, admixtures, etc.) and field uncertainties make concrete susceptible to imprecise determinations of strength in the early stages of curing; managing these factors is important in preventing structures from unexpected accident or failure due to the premature removal of a framework or immature concrete. It is necessary to understand the materials used for construction to ensure the efficiency and longevity of the structures being built. Several types of structures are built using concrete. It is important to study and determine the properties of cement and concrete at different stages of construction as they directly affect the strength and durability of the structures. In the project schedule for construction, the idle time required for the hardening of concrete is a crucial influencing factor, especially in construction of high rise or multi-staged projects. Unscheduled removal or early removal of the supportive framework sometimes causes huge losses, such as the incident that took place in the construction of 2000 Commonwealth Avenue, Boston [1]; one side of the building progressively fell down, causing the death of four workers and injury to twenty others. Later, it was reported that the collapse was initiated due to a roof slab that was insufficiently cured due to exposure to cold weather. Structures such as high-rise buildings, dams, and bridges need precise monitoring in the early stages of construction because the size of the structures becomes a challenge for safety and quality assurance. For the safe loading of structural elements, applying pre-stressing, removing form work, and limiting unnecessary time between project stages, it is necessary to understand the curing process and be familiar with concrete strength and stiffness. Monitoring of the curing and strength development processes provides enough information for these stages. Consequently, for quality assurance, health monitoring, and safety control, many techniques of destructive and non-destructive testing (NDT) are used [2,3,4,5,6,7,8,9,10,11,12,13]. The innovations in this field have led the way to smart construction, and utilizing the idea of the IoT (Internet of Things) has upgraded the structural health monitoring process to precisely and remotely monitor the structures and to provide a safe work environment. These innovations tie the IoT to data simplification for destructive and NDT [2]. Conventionally, compressive strength tests of concrete use concrete cylinders or cubes cast along the structure. Even though this technique is simple and economical, various factors cause a difference in the strength of the main structure and of these cubes; one of those factors is spatial variation. On the other hand, NDT is preferred as it is more practical, quick, and cost effective. Some of the NDT techniques that are widely used are the ultrasonic pulse velocity, Schmidt rebound hammer test, radiography, resonant frequency, isothermal calorimeter, maturity method, and some acoustic tests [14,15,16,17,18,19,20,21,22,23,24]. The traditional method for measuring concrete strength may need adjustments of special equipment and to be carried out during construction, which can interrupt the tough construction schedule, while the smart techniques do not delay construction and can provide data for as long it is needed in the process of structural health monitoring (SHM). The accuracy of the results for these tests differs from case-to-case, depending on the technique used. The ultrasonic waves analysis, which is based on a resonant frequency change and uses different features of wave propagation, is the test with enough data availability for analysis. The application of ultrasonic waves propagation is widely used for structural health monitoring, concrete compressive strength prediction, and damage detection in structures [9,25,26]. The technique generously reveals elastic and dynamic properties of the concrete, making this procedure plausible for concrete structural health monitoring using piezoelectric (PZT) materials [6,8,26,27,28]. In ultrasonic waves analysis, PZT sensors are used to mechanically measure the dynamic responses of the ultrasonic waves that display boundary disparities owing to the development of concrete strength. The velocities of the waves are determined from the arrival time of the signals. Because the PZT materials are used as sensors and actuators, the distance between the actuator and sensor with the arrival of the first return signal is used to modify the propagation process. For fresh concrete, the approach of the ToF (time-of-flight) to the arrival of the first signal gives smooth results; monitoring of velocities for the P-waves and R-waves gives extensive data about the concrete [8,9]. For these approaches, the applied simplification differs for the surface and internal waves. The shared waves may also be used, giving adequate results for damage and crack detection. Simultaneously, embedded, wireless temperature sensors measure the hydration temperature history of the concrete to estimate the concrete early-age strength using the maturity method [16,17,29,30]. The maturity theory states that the graphical area of temperature experienced by a specimen, beyond the datum temperature of the concrete, predicts the concrete strength by using the Nurse–Saul maturity index. In this case, the hydration temperature and the curing age forecast the strength of the concrete on a formulated basis [29,31]. The ASTM (American Society for Testing and Materials) provides two maturity functions for the maturity tests: (i) the Nurse–Saul function and (ii) the Arrhenius function, which uses the temperature history of hydration and the activation energies of the cementation process [16]. In this maturity method study, temperature effects were analyzed for the strength-gaining process and the effects of hydration temperature were determined for early-age concrete strength development. In this procedure, we acquired the data by wireless sensors embedded in the concrete, processed the data for noise reduction, and used the Nurse–Saul method of maturity curve development. For competence of the data and to streamline the work, the results were compared with those formulated by maturity method data, ultrasonic wave propagation results, and the compressive strength gained by a penetration resistance test using a universal testing machine (UTM) and conducted on cylindrical specimens taken from the same concrete mix.

## 2. Transition of Pattern, Dissimilation of Signals

### 2.1. Waves Direct Velocities

Ultrasonic wave propagation through the concrete medium is studied widely. The variations of these approaches lay in the methodology used for analyzing the wave propagation. For this purpose, different parameters are analyzed related to concrete stiffness and compressive strength properties. Normally the technique of direct transmission of waves through the concrete medium is used. By using the ultrasonic waves analysis method, three types of data can be acquired when dynamic loads or vibrations are applied to a concrete structure: (i) compressional waves or P-waves, (ii) Rayleigh or surface waves, and (iii) shear waves or S-waves [32,33]. The characteristics of these waves can be determined by the patterns that depend on the velocity, amplitude, and the relationship of these wave parameters. Transmission of compressional waves have high velocity whereas the velocities of shear and surface waves are approximately 60% and 55% of that of compressional waves, respectively. The relationship between the concrete strength and waves is usually given by [34],

(1) fc=a exp(b V).

In this equation *fc* represents the compressive strength, *V* is the velocity of the longitudinal waves, and a and b are the parameters determined by P. Turgut [35] using the least squares method. The quality monitoring and assurance by wave propagations follows the theory of kinematics of deformation, which is based on Newton’s second law of motion [36]. According to this theory, the total stresses acting on the subjected area of a concrete specimen is the second order differential governing longitudinal wave propagation,

(2) $$\frac{\partial^{2}u}{\partial t^{2}}=S^{2}\frac{\partial^{2}u}{\partial x^{2}}.$$

In Equation (2), *S* is the speed of the wave, u is the displacement, *t* represents the time, and *x* is the path length.

For measurement of stressed zones, the waves speed can be used. The wave speed depends on the density and Young’s modulus according to,

(3) $$S=\sqrt{\frac{E}{\rho_{o}}}.$$

In Equation (3), Young’s modulus and the density of the material are represented by *E* and $\rho_{o}$ respectively. In stress waves, the Poisson ratio of the material affects the velocity, so the effects are countered using Equation (4) [35],

(4) $$S=\sqrt{\frac{E}{\rho_{o}}}\frac{1-v}{\left(1+v\right)\left(1-2v\right)}.$$

In this equation, *v* is the Poisson ratio of the material, which is incorporated to give the value for the speed of the waves in the material.

### 2.2. Molded Variation

Because the stiffness and rigidity grow with the curing time of the concrete, the velocity of the waves increases as a function of time. This variation in stiffness molds the velocity of the waves. The stiffness of the material is related to the Young’s modulus of material [37]. Thus, with curing time, the Young’s modulus also rises.

According to the wave equation, the relation of wave speed, Young’s Modulus, and density of material is defined as,

(5) $$\frac{\partial^{2}u}{\partial t^{2}}=\frac{E}{\rho_{o}}\;\frac{\partial^{2}u}{\partial x^{2}}=S_{1}^{2}\frac{\partial^{2}u}{\partial x^{2}}.$$

In Equation (5), *E* is Young’s modulus, $S_{1}$ is the speed of the elastic wave, u is the displacement, *t* is the time, *x* is the path length, and $\rho_{o}$ is the density of the material in its unstrained state.

## 3. Experimental Setup

This study of concrete comparative strength-development monitoring was prepared using three different results: (i) the maturity method of concrete strength estimation, (ii) ultrasonic wave propagation in concrete, and (iii) UTM-based concrete compressive strength estimation, which was composed of smart PZT sensors and temperature sensors (SmartRock) to monitor the commencement of dynamical changes in the concrete specimens.

### 3.1. Mixture Composition and Material Gradations

For the experiment, a cubic meter sample composition was prepared according to quantities given in Table 1. For the admixture, silica fume was added for bond strength and compressive-strength gain. The machine mix of cement and aggregates was prepared, and the admixture was added before adding water.

**Figure 1:**
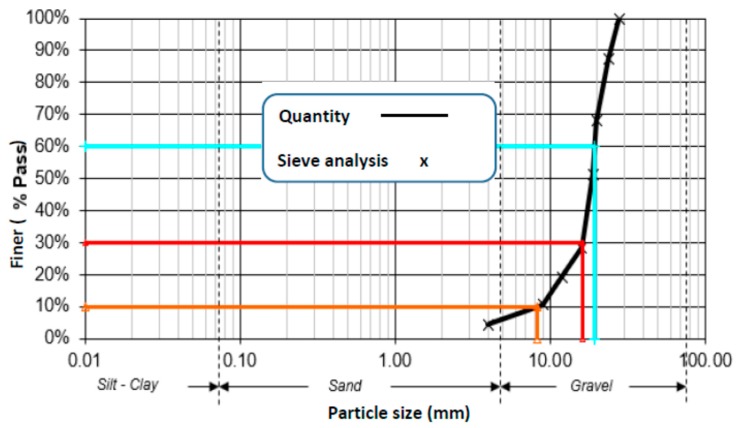
Gradation of gravel size for the concrete mixture.

Ordinary Portland cement of type ASTM C 150 was used in the preparation of the specimens. The gradation of aggregates, determined by sieve analysis, is given in Figure 1. Sand was used for fine aggregates and crushed stones were used for coarse aggregates. The size of the coarse aggregates used was 18 mm.

### 3.2. Specimen Properties

Concrete specimens of dimensions 400 mm × 100 mm × 100 mm were casted for the test of ultrasonic waves and maturity method strength monitoring. The PZT transducers and temperature sensors were embedded in the specimens during pouring of the concrete. Simultaneously, cylindrical samples were cast for the compressive strength test by penetration resistance using a UTM from the same concrete mixture. Two types of specimens of the same concrete mix were poured for the experiment, the specimens of which dimensions are aforementioned were used for the ultrasonic wave propagation monitoring and SmartRock sensors. The cylindrical samples were poured for the destructive test to be placed as part of the penetration resistance test. The average value of three specimens was considered the value for each specific day during the strength-development monitoring; monitoring was completed on days 1, 2, 3, 7, 14, and 28.

### 3.3. PZT Material as Sensors and Actuators

The PZT material has the ability to interconvert mechanical and electrical energies and sense the dynamical vibration in paste and solid media. These rich properties enable PZT materials to be used both as sensors and actuators [14,38,39,40]. As the PZT materials are used as transducers, for the needs of sensing and actuating, the terms of PZT sensors and PZT actuators are used, respectively, according to placement. Researchers have employed these materials using different approaches as sensors. PZT sensors can be used to extract different mechanical properties of the subject samples or structures. Furthermore, using PZT materials for different approaches and to consider various parameters can determine strain, stiffness, and stresses, or other modified features of material. The strain effects and electro-mechanical effects of the PZT sensors are used to investigate the dynamical properties of the host structure. In this study, the property of propagation of ultrasonic waves was utilized to study and analyze the dynamic changes observed with the curing age and development of concrete strength [8,9,10,41,42].

### 3.4. Sensors’ Preparation

**Figure 2:**
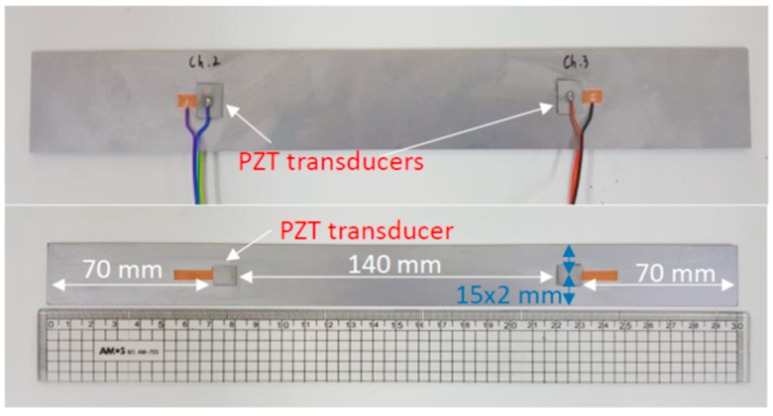
Piezoelectric (PZT) transducer locations on plate, including dimensions.

For data acquisition, PZT materials were used to actuate and sense the responses of propagated signals from the medium. The specific PZT material used in this experiment was APC 850 (American Piezo Ceramics), which has high sensitivity characteristics and large displacement effect; these PZT transducers were embedded in the concrete with large damping effects. The material of size 10 mm × 10 mm was attached to 300 mm × 30 mm steel plates such that two PZT specimens were placed on each face of a steel plate separated by 140 mm (a total of four transducers on a single steel plate, PZT specimens were attached on both sides of the plates), as shown in Figure 2. Here, the size of the specimen is smaller than the targeted structure, and further resizing can take place according to the needs and propagation plan. Because the thick transducers generated the ultrasonic waves in the direction of the thickness of the steel plates, thin PZT transducers were used to generate guided waves along the surface of the steel plates. The distance between pairs of the PZT transducers were determined such that signals from the direct path of the PZT transducers could not contaminate the reflected signals from the side and end boundaries of the steel plate.

**Figure 3:**
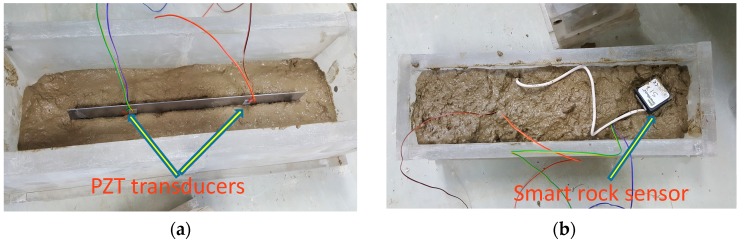
(**a**) Embedded PZT transducers in concrete specimen and (**b**) allocation of SmartRock (temperature sensor).

The PZT transducers were embedded in concrete specimens to actuate and produce guided ultrasonic waves and were considered as a sensor to observe the internal dynamic changes of the subjected material [39]. The sensors were embedded in the specimen during the pouring of the concrete and positioned to keep the connections safe for acquiring the data as shown in Figure 3a,b.

Because the electrical behavior of the material is linear,

(6) $$D_{i}=\varepsilon_{ij}E_{i}.$$

In Equation (6), *D* is the displacement of the electric charge density, *E* is the electric field strength, and *ε* is the permittivity of a free body. The factor of proportionality relating the electric field strength and electric displacement is defined as the medium’s dielectric constant. The tensor of the displacement of the electric charge density is also represented in Equation (6).

The strain–stress relationship is in accordance with Hooke’s Law,

(7) $$S_{i}=S_{ij}T_{j}.$$

In this equation, *S* represents the strain and *T* represents the stress produced in the body.

Equations (6) and (7) can be written as

(8) $$S=s^{E}T+dE,\;D=dT+\varepsilon^{T}E.$$

In Equation (8), $s^{E}$ is the compliance under the electric field and $\varepsilon^{T}$ represents the dielectric constant of the PZT material under constant stress. Equation (8) is used to simplify the stress and strain effects occurring in the body.

### 3.5. Methodology Analysis for the Longitudinal Wave Variations

The approach for conducting experiments for this study encompassed embedding smart PZT transducers in a concrete specimen to measure the ultrasonic waves variation of center frequencies in the range of 100–300 kHz and thus to monitor the hardening process of concrete by the gradual divergence of phase-transition as the specimens cured. Especially in the liquid phase to the initial sitting time, the strength measuring process is critical, in that it depends on the mixture type and sometimes varies with the measuring approaches. In the initial sitting time, the frequencies of waves indicate the dynamic change in the medium. In this study, we monitored the phase changes of the A-mode and S-mode of signals.

The experiment was carried out with a concrete mixture ratio given in Table 1 and kept at a temperature of 23 °C. The data from the samples were collected continually every thirty minutes, with the aid of a computer setup. For the measurement of data, a setup of an arbitrary waveform generator (AWG), controller, multiplexers (MUX), and a signal digitizer (DIG) was assembled to acquire and process the data. The ultrasonic wave velocity profiles were obtained as a function of time. In this experiment, the velocity variation was studied to estimate the compressive strength of concrete. With the gradual change in the amplitude of the ultrasonic waves, the change of strength became more prominent and was easier to observe and calculate. For this purpose, the changes in the pattern were approached differently from the Roa-Rodriguez method [43].

In the process of ultrasonic wave propagation, to consider the analogy of phase transition, the simplified one-dimensional equation fits adequately because the ultrasonic pulse detector or receiver focuses only on the first arrival of the signal of the longitudinal wave and the subsequent signals are ignored by the system. Furthermore, ultrasonic waves were analyzed through the functionality of the solid-medium wave-transformation analogy. Studies of the phase transition state that for the monitoring of composite structures, the A-mode of Lamb waves gives a clearer and more appropriate result than the S-mode [44,45]. When the propagated wave travels in-phase, it is considered as S-mode and when it travels out-of-phase it is considered A-mode. The relationship can be altered by the placement of the PZT transducers and the polarization of the specimens. This makes it easy to recognize the individual modes of the waves by adding or subtracting one signal from the other. As for the nomination of the PZT transducers, the arrangement of the signals can be made according to its order. If cracks or damage are detected, the signal’s phases will be identical. The waves propagation speed, s, in Equation (5) is independent of ∂*u*/∂*t*, or that of the local velocity/speed shift of the elements transmitting the wave through sections. The time-of-flight is used for the specification of the velocity change of the waves. The velocity of waves is modified by dynamic variations of the material thickness. The elastic properties of the transmuting medium, density, and Poisson’s ratio define the wave speed, s. In this study, all the wave speed values are assumed constant. Hence, the results were compared for all three methods.

## 4. Hydration Temperature and Maturity Index

The hydration temperature history uses data for the NDT to reveal hydration-temperature-related features to monitor compressive strength of fresh concrete. This method relies on the combined parameters of temperature and time and can be used for in-place concrete during construction [17,46].

**Figure 4:**
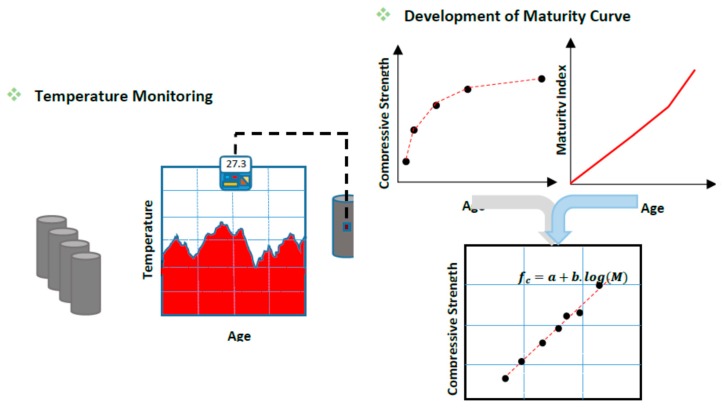
Schematic procedure of temperature history monitoring, maturity index, and compressive strength in the concrete maturity method.

When water is added to the mixture, the hydration process commences, and the chemical reaction of disilicate, trisilicate, aluminate, and other admixtures begins, so the hydration temperature of concrete rapidly increases. The temperature rises rapidly during the initial hours of pouring until two days later, after which it stays constant with the development of the material’s internal bonding and increased concrete strength [47]. In the maturity method depicted in Figure 4, after pouring concrete, the hydration temperature is the key feature to determine the maturity curve and estimate the maturity function for the prediction of concrete strength. For the strength development, the chemical bonding of individual particles is linear. The related study of N.J. Carino states, in Equation (9), that the shrinkage of chemical bonding is a dependent function of age and gives a clear result for the hydration temperature [17]. The strength can be further clarified by considering the datum temperature as related to the mixture type,

(9) $$S=S_{u}=\frac{\sqrt{k\left(t-t_{0}\right)}}{1+\sqrt{k\left(t-t_{0}\right)}.}$$

In this equation, S represents the strength with respect to time *t*, *t*_0_ is the time at initial sitting, and $S_{u}$ represents the limiting strength of the sample. In Equation (9), *k* is a constant rate, which in a molecular system is when the energy is transferred between the molecules due to collisions during their constant motion. The kinetic energy of the molecules increases as the system heats up and molecules surmount the barrier of energy required for lower-energy reaction products. Thus, the rate of reaction increases with increasing temperature observed as a constant rate *k* [48].

While we can define the datum temperature of the mixture according to the properties of cement and other admixtures, when admixtures are added, the datum temperature can be different. In slack seasons of concrete pouring, freezing-temperature effects on the strength-gaining process of inter-particle bonding must be considered when specifying the datum temperature [17].

The product of time and hydration temperature of concrete is used for strength estimation. The 1950s Nurse–Saul maturity Function is given in Equation (10) [29]

(10) $$M=\overset{t}{\underset{0}{\sum }}\left(T-T_{0}\right)\Delta t.$$

Equation (10) shows the datum temperature effect is neglected to prominently pursue the strength development.

## 5. Discussion and Results

### 5.1. Phase Variation

In monitoring the strength-development process of concrete through ultrasonic wave analysis, the dynamic change of the phase in the material observed for the initial sitting stages used the phase-transition phenomena. At the same time, the hydration-temperature history produced the maturity curve to predict the strength using the maturity index. The comparison of these results was established by measuring the strength of the cylindrical simples by the penetration resistance test using a UTM. The results of the maturity method and the compressive strength were clear and similar to the ultrasonic waves analysis result.

**Figure 5:**
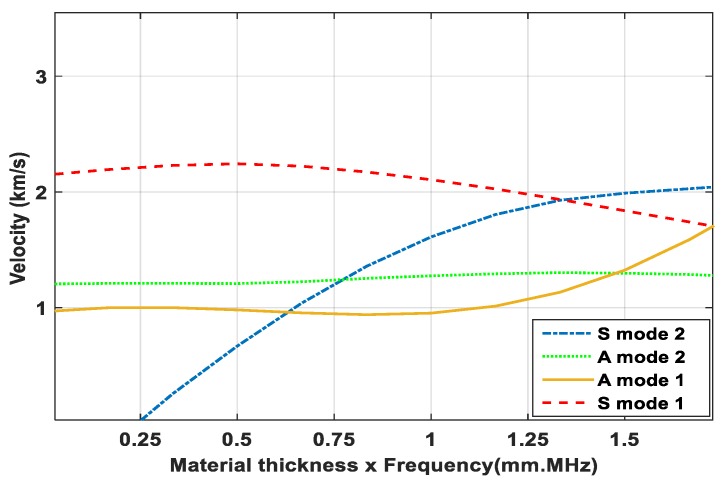
Wave modes for the arrival time of signals.

The velocity variations in the wave propagation were observed in the transmuting of phases of the concrete sample. The phase changes were due to the formation of new boundary conditions with curing and strength development, as the strength of the inter-particle bonding caused the expansion in the boundaries of the concrete structure. The velocity change of A-mode compared to S-mode for the specimens is shown in Figure 5. The fast-changing arrival time of signals for the A-mode defines the boundary condition, the S-mode waves change later with the changing of the boundary conditions. The approach of using the first signal observed by the sensor defines the thickness of the material of the transmitted wave, which deals with the signal’s travel time between two specific points.

**Figure 6:**
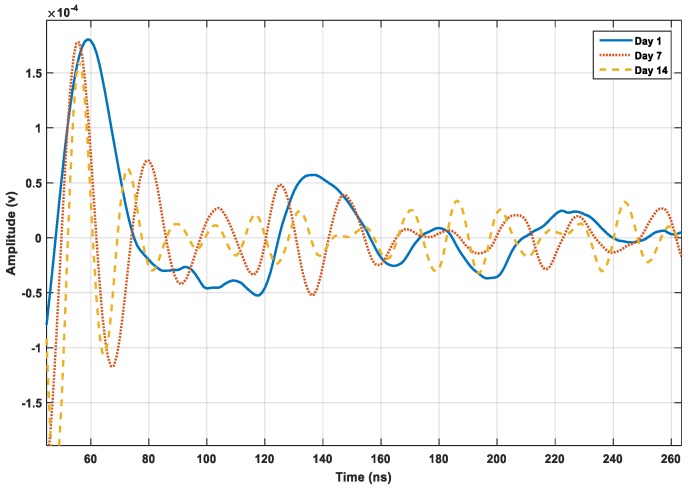
Raw signals amplitude variation on days 1, 7, and 14.

Pattern irrelevancy in the raw signals can be observed for the amplitude change in Figure 6, which shows the data of three different curing ages. Due to phase regulation in the strength gain, there is a clear difference among the data. The arbitrary waveform generator emits ±10 signals peak-to-peak with a frequency ranging from 100 to 300 kHz; the frequency is exerted by the PZT transducers to form the signals. Figure 6 shows the data of the raw signals for different periods. The peaks of the signals with initial generation time have high amplitudes, which decrease with time. The peak inclination differs for different periods and indicates the dynamic variations.

**Figure 7:**
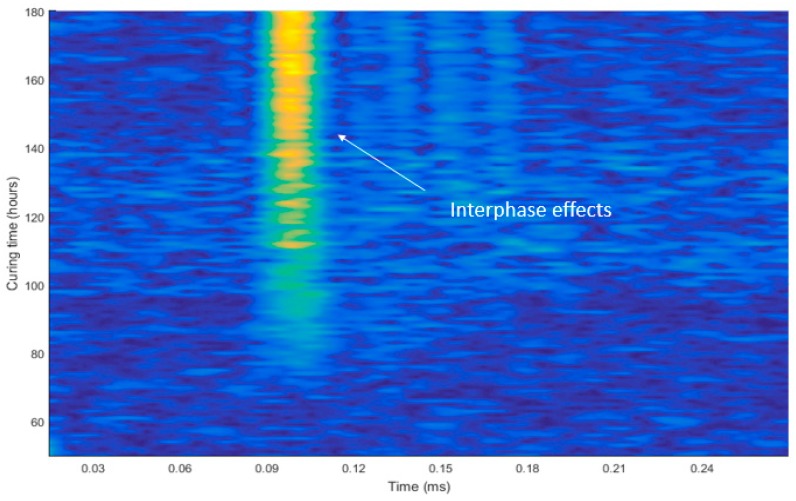
Phase-change response in signals propagation.

The velocity change in the term of the material stiffness factor occurs prominently in Figure 7; the deepness of the color indicates the rapidity in the velocities and changes of the phases in the material. The wave propagation with curing time changes as the stiffness of the material changes. As the velocities of signals at frequencies near 100 kHz are selected for the A-mode waves, the lower noise data can predict the speed of the waves as a factor for strength monitoring.

### 5.2. Hydration Temperature and Maturity Index

**Figure 8:**
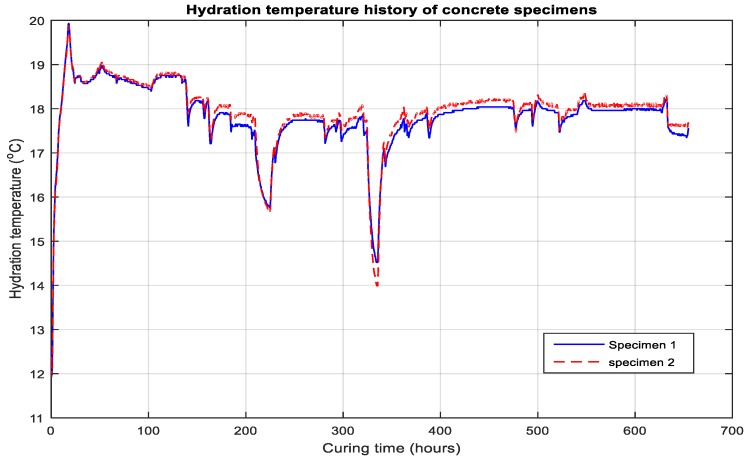
Hydration temperature for concrete specimens 1 and 2.

The hydration temperature was monitored continuously after pouring the concrete and the smart temperature sensors produced the data for the estimation of the maturity of concrete with curing time. The datum temperature was calculated as −2 °C per ASTM [16,17]. The data of the temperature for the hydration heat is shown in Figure 8.

**Figure 9:**
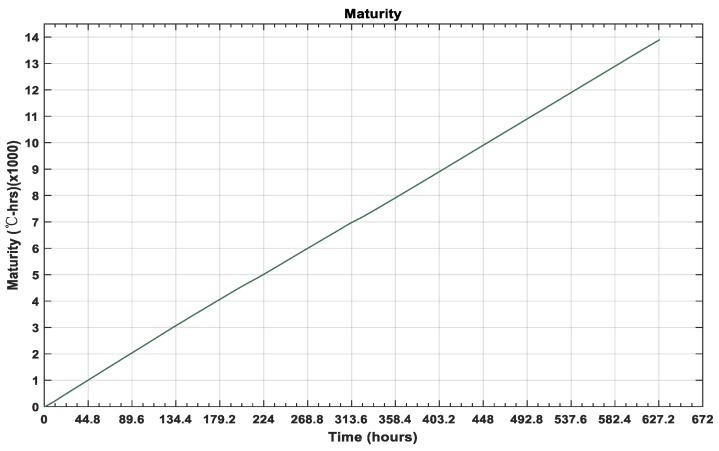
Maturity data for the hydration-temperature monitoring.

The data of hydration-temperature history was applied to the Nurse–Saul curing function to predict the maturity index. As the datum temperature and time limit were defined, the results showed that hydration temperature was elevated in the initial hours and increased rapidly; that elevation continued to be observed until 28 h after curing, reaching a rise of 20 °C. As time passed, the strength-development improved and the temperature was observed to show lower peaks, as compared with the initial hours, owing to the completion of chemical processes. The experiment was composed of two specimens in the same environmental conditions. Consequently, the temperature data did not show a prominent deviation in curves. The data for the maturity with curing time is shown in Figure 9.

**Figure 10:**
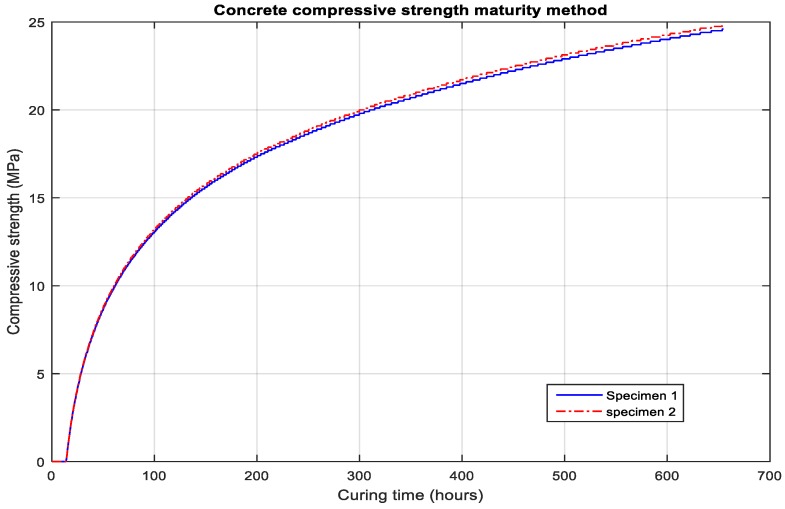
Concrete compressive strength data from temperature sensors.

Because the temperature data for the concrete specimen 2 appeared slightly higher than that of specimen 1 of concrete, the cured strength was also calculated to be somewhat higher. The strength was calculated from the data shown in Figure 10.

Using the Nurse–Saul function with the datum temperature measured as −2 °C, the maturity index data were calculated as shown in Figure 9, which further simplified the strength of the concrete specimen. The rapid strength development at early ages is prominent from the data results and the compressive strength at the targeted time measured 24.47 MPa, as shown in Figure 10.

### 5.3. Comparative Data for the Strength Prediction

**Figure 11:**
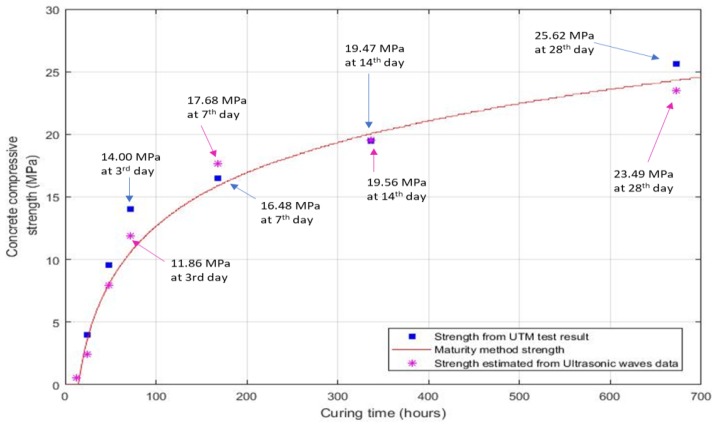
Concrete compressive strength measured by the maturity method, universal testing machine (UTM) test, and ultrasonic wave data.

The results of compressive strengths were compared to the penetration test results performed in the laboratory on the specimens for penetration-resistance tests. The data for all indicated dates were the average values of the results for the three cylindrical-specimen tests on each date. After 24 h of curing, the average strength of three cubes for the penetration resistance test was calculated as 3.97 MPa, which rapidly increased until 48 h when the strength value reached 9.58 MPa, as shown in Figure 11. The gain in the strength curve of the ultrasonic waves data and the maturity results was observed to be relatively slow, owing to the specimen size effect. At the third day of curing, the concrete strength approached 11.68 MPa and 14.00 MPa for the ultrasonic waves data and the cylindrical samples penetration test, respectively, while the maturity method yielded a result of 10.92 MPa.

**Figure 12:**
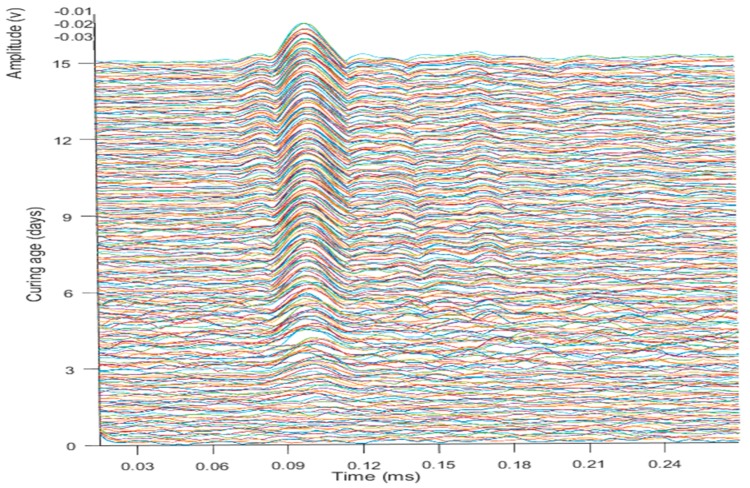
Amplitude rise pattern with curing time for ultrasonic waves data.

The variations in the results can be observed by the data, which was due to categorizing the rise of amplitudes in the data of the ultrasonic wave signals in Figure 12. Variation of the waves becomes higher and more prominent with increasing curing time due to the gaining of strength by the concrete specimen and the strengthening of the cohesive power of the concrete particles, which defines the limitations of the concrete material. The propagation of the signals becomes more dominant with time.

The strength prediction for the described methods produced a simultaneous curing process for the gain of strength, and the results were calculated as 16.48 MPa and 17.68 MPa for the UTM test and ultrasonic wave analysis, respectively.

The deviation of the data and specification of the strength-development monitoring is compared by fitting a curve to the analyzed data of the ultrasonic waves propagation data and the maturity method results to make the comparison with compressive strength measured by the penetration resistance test. The fitness of regression for the data model was 0.997, implying a valid fit and permitting a novel modified relation for predicting ultrasonic wave velocities in concrete and the maturity of the concrete at an early age. This is specified in Equation (11),

(11) $$C^{\prime }\left(v,m\right)=9.903v+0.000599m-22.72.$$

**Figure 13:**
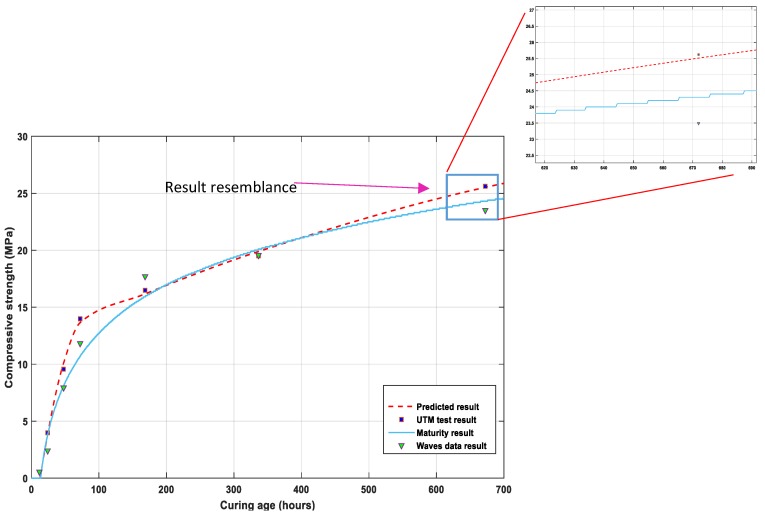
Data comparison for the concrete compressive strength with the maturity- and ultrasonic velocities-modified data.

Here, $C^{\prime }$ represents the compressive strength calculated from the penetration test data, *v* represents the wave propagated data, and *m* is the maturity method result for the estimated compressive strength. The equation provides a promising result for all the data and is in good agreement with the data shown in Figure 13.

The experiments were performed with a controlled temperature, but in addition to temperature, other factors like water/cement ratio, type of bonding agents, aggregates, and age of concrete may affect the concrete-strength process [47,49]. As shown in Figure 11, the strength calculated from the data of hydration temperature differs by 0.86 MPa from the strength gain measured by a UTM test; this is due to the spatial variation effect of the specimens. The effect of concrete compactness was same in the uniform samples.

On day 14, the average strength of the penetration test was calculated as 19.47 MPa and that of the waves analysis was measured as 19.56 MPa, nearly the same for both specimens, while the result of the maturity method was calculated as 20.02 MPa, as shown in Figure 13. The results of the concrete specimens at day 28 were higher: 25.62 MPa for the penetration resistance test of the cylinders, 24.13 MPa for the data of concrete maturity method, and 23.49 MPa for the waves data, which provided a clear demonstration of the test results for the study.

This modified new relationship of maturity and ultrasonic waves data results predict a comparative function for estimation in-situ of concrete early-age strength by NDT methods, which is a novel approach for early-age strength estimation. Concrete strength at day 28 by the destructive test of penetration resistance of concrete cubes through a UTM had the strength of 25.62 MPa, while the result according to the equation calculated the strength as 25.50 MPa at day 28. This relationship of data in the study attempts to predict the compressive strength by different means, leading to safe, promising structural health monitoring.

## 6. Conclusions

This study covers the limitations of early-age strength development of concrete through an analysis that incorporates different test methodologies. The relationship of the maturity method and the ultrasonic wave propagation is that the hydration temperature results become higher in the initial hours of the hydration process; the wave propagation is slow as the concrete mix paste changes from a liquid paste to a semisolid form, the observed fluctuation of the waves was due to the medium form variation. The results predicted by the data, illustrated in Figure 13, are promising for concrete early-age strength monitoring and strength development estimation. The ultrasonic wave-propagation experiment provided a massive amount of data for predicting the compressive strength of undisturbed field-poured concrete as an NDT function, yielding a modified equation that gives a comparative result for strength estimation, which allows engineers a quick and safe project implementation process.

The data suggest that the behavior of the signals and time of wave propagation to the boundary of the higher rigidity in the concrete sample results in a higher value of the wave propagation velocity.

For the maturity of concrete, the datum temperature plays a basic role in the results of the maturity index, as the maturity method result gave a smooth output and was a little higher than that of the ultrasonic waves. So, for each mix, the datum temperature should be defined according to mix proportions, as the hydration temperature directly affects the strength development. Furthermore, from the results, it has been concluded that high temperature during concrete curing causes rapid strength gains in the structural elements.

The results qualify the proposed method for structural health monitoring in a wide range of industrial multi-stepped project monitoring. The procedure can be advanced in the future for the integration of methodology to the equipped systems for synthesis in the modified polymers.

## Figures and Tables

**Table 1:** Concrete mix details (kg/m^3^).

Materials	Cement	Silica Fume	Silica Powder	Sand	Aggregates	Water	Fiber (Vol %)
Quantity	330	0.165 (0.05%)	2.310 (0.70%)	873	916	165	0

## Abbreviations

NDT	non-destructive testing
UTM	universal testing machine
RC	reinforced concrete
AWG	arbitrary waveform generator
MUX	multiplexer
SHM	structural health monitoring
PZT	piezoelectric
ASTM	American Society for Testing and Materials
APC	American Piezo Ceramics
IoT	Internet of Things

## References

[1] King S., Delatte N.J. (2004). Collapse of 2000 Commonwealth Avenue: Punching shear case study. J. Perform. Constr. Facil..

[2] Zinno R., Artese S., Clausi G., Magaro F., Meduri S., Miceli A., Venneri A. (2019). Structural health monitoring (SHM). The Internet of Things for Smart Urban Ecosystem.

[3] Sýkora M., Diamantidis D., Markova J., Rózsás Á. (2018). Assessment of compressive strength of historic masonry using non-destructive and destructive techniques. Constr. Build. Mater..

[4] James H., Massoud S., Priyan M. (2015). Non-Destructive Testing of Concrete: A Review of Methods. Electron. J. Struct. Eng..

[5] Wahab A., Aziz M., Rahman A., Sam M., Qadir A., Kassim K., You Y. (2019). Review on microwave nondestructive testing techniques and its applications in concrete technology. Constr. Build. Mater..

[6] Gu H., Song G., Dhonde H., Mo Y.L., Yan S. (2006). Concrete early-age strength monitoring using embedded piezoelectric transducers. Smart Mater. Struct..

[7] (2002). Guidebook on Non-Destructive Testing of Concrete Structures.

[8] Lim Y.Y., Kwong K.Z., Liew W.Y.H., Chee K.S. (2016). Non-destructive concrete strength evaluation using smart piezoelectric transducer—A comparative study. Smart Mater. Struct..

[9] Cédric D., Grigoris K., Jérôme C., Stéphanie S., Arnaud D. (2012). Monitoring of the ultrasonic P-wave velocity in early-age concrete with embedded piezoelectric transducers. Smart Mater. Struct..

[10] Sung W.S., Adeel R.Q., Jae Y.L., Chung B.Y. (2008). Piezoelectric sensor based nondestructive active monitoring of strength gain in concrete. Smart Mater. Struct..

[11] Kim J.W., Park S.H. (2018). MFL sensing and ANN pattern recognition based automated damage detection and quantification for wire rope NDE. Sensors.

[12] Kim J., Kim J.W., Lee C., Park S. (2017). Development of Embedded EM Sensors for Estimating Tensile Forces of PSC Girder Bridges. Sensors.

[13] Oh T.K., Kim J., Lee C., Park S. (2017). Nondestructive Concrete Strength Estimation based on Electro-Mechanical Impedance with Artificial Neural Network. J. Adv. Concr. Technol..

[14] Constantin E.C., Chris G.K., Georgia M.A., Nikos A.P., Maria J.F., Costas P.P. (2016). Application of smart piezoelectric materials in a wireless admittance monitoring system. (WiAMS) to structure—Test in RC elements. Case Stud. Constr. Mater..

[15] Priya B., Thiyagarajan J., Monica B., Gopalakrishnan N., Rao A. (2018). EMI based monitoring of early-age characteristics of concrete and comparison of serial/parallel multi-sensing technique. Constr. Build. Mater..

[16] ASTM International (2004). Standard Practice for Estimation Concrete Strength by the Maturity Method. ASTM C.

[17] Carino N.J., Lew H.S. The maturity method: From theory to application. Proceedings of the 2001 Structures Congress & Exposition.

[18] Blaschke J.H.V., Torrico F.A. (2018). Estimaing concrete strength using the correlation of the concrete maturity method applied to the mattrials of Cochabamba-Bolivia. Rev. Investig. Desarro..

[19] Malhotra V.M., Carino N.J. (2003). Handbook on Nondestructive Testing of Concrete Second Edition.

[20] Bungey J.H., Grantham M.G. (2014). Testing of Concrete in Structures.

[21] Park S., Kim J.-W., Lee C., Park S.-K. (2011). Impedance-based wireless Debonding condition monitoring of CFRP Laminated Concrete structure. NDT E Int..

[22] Costas P.P., Stavros E.T., Evangelos V.L. (2018). 2-D Statistical Damage Detection of Concrete Structures Combining Smart Piezoelectric Materials and Scanning Laser Doppler Vibrometry. SDHM.

[23] Reinhardt H.W., Grosse C.U. (2004). Continuous monitoring of setting and hardening of mortar and concrete. Constr. Build. Mater..

[24] Bhalla S., Soh C.K. (2004). Structural health monitoring by piezo-impedance transducers. J. Aerosp. Eng..

[25] Hyejin Y., Kim J.Y., Kim S.H., Kang W.J., Hyun K.M. (2017). Evaluation of Early-Age Concrete Compressive Strength with Ultrasonic Sensors. Sensors.

[26] Lim Y.Y., Tang Z.S., Smith S. (2018). Piezoelectric based monitoring of structural adhesives curing: A novel experimental study. Smart Mater. Struct..

[27] Hudson T.B., Auwaijan N., Yuan F.G. (2018). Guided wave-based system for real-time cure monitoring of composites using piezoelectric discs and phase-shifted fiber Bragg gratings. J. Compos. Mater..

[28] Tavossi H.M., Tittmann B.R., Cohen T.F. (1999). Ultrasonic characterization of cement and concrete. Review of Progress in Quantitative Nondestructive Evolution.

[29] Marios S., Fragkoulis K. (2018). The modified nurse-saul (MNS) maturity function for improved strength estimates at elevated curing temperatures. Case Stud. Constr. Mater..

[30] Marcus P., Gregorz F., Pawel N., Tim R., Jack M. (2017). Wireless Concrete Strength Monitoring of Wind Turbine Foundations. Sensors.

[31] Ramachandran V.S., James J.B. (2000). Handbook of Analytical Techniques in Concrete Science and Technology.

[32] Lee Y.H., Oh T. (2016). The Measurement of P-, S-, and R-Wave Velocities to Evaluate the Condition of Reinforced and Prestressed Concrete Slabs. Adv. Mater. Sci. Eng..

[33] Hall K.S. (2011). Air-Coupled Ultrasonic Tomographic Imaging of Concrete Element. Ph.D. Thesis.

[34] Trtnik G., Kavcic F., Turk G. (2008). Prediction of concrete strength using ultrasonic pulse velocity and artificial neural networks. Ultrasonics.

[35] Turgut P. Evaluation of the ultrasonic pulse velocity data coming on the field. Proceedings of the Fourth International Conference on NDE in Relation to Structural Integrity for Nuclear and Pressurized Components 2004, Session B.

[36] Jian H.Y., Yuan J.-X., Liu M.-G. (1999). Assessment of pil integrity by low strain stress wave method. HKIE Trans..

[37] David R. (2008). Mechanical Properties of Materials. http://web.mit.edu/course/3/3.225/book.pdf.

[38] Kim J.W., Kim J.K., Park S.H., Oh T.K. (2015). Integrating embedded piezoelectric sensors with continuous wavelet transforms for real-time concrete curing strength monitoring. Struct. Infrastruct. Eng..

[39] Maeder M., Damjanovic D., Setter N. (2004). Lead Free Piezoelectric Materials. J. Electroceram..

[40] Elena A., Jacob L.J. (2010). Advances in Lead-Free Piezoelectric Materials for Sensors and Actuators. Sensors.

[41] Yunzhu C., Xingwei X. (2018). Advances in the structural health monitoring of bridges using piezoelectric transducers. Sensors.

[42] Guoqi Z., Di Z., Lu Z., Ben W. (2018). Detection of Defects in Reinforced Concrete Structures Using Ultrasonic Nondestructive Evaluation with Piezoceramic Transducers and the Time Reversal Method. Sensors.

[43] Roa R.G., Aperador W., Delgado A. (2013). Simulation of Non-destructive Testing Methods of Ultrasound in Concrete Columns. Int. J. Electrochem. Sci..

[44] Zailin Y., Hamada M.E., Yao W. (2014). Damage Detection Using Lamb Waves (Review). Adv. Mater. Res..

[45] Marilyne P., Constantinos S., Matthieu G., Kui Y. (2018). Damage Detection in a Composite T-Joint Using Guided Lamb Waves. Aerospace.

[46] (2018). Concrete in Practice. National Ready Mixed Concrete Association.

[47] Taylor P.C. (2013). Curing Concrete.

[48] Brown T.L., LeMay H.E. (1988). Chemistry: The Central Science.

[49] Rose K., Hope B.B., Ip A.K.C. (1989). Factors affecting strength and durability of concrete made with various cements. Transp. Res. Rec..

